# Analysis of mRNA recognition by human thymidylate synthase

**DOI:** 10.1042/BSR20140137

**Published:** 2014-12-23

**Authors:** Nicholas D. Brunn, Sergey M. Dibrov, Melody B. Kao, Majid Ghassemian, Thomas Hermann

**Affiliations:** *Department of Chemistry and Biochemistry, University of California, San Diego, 9500 Gilman Drive, La Jolla, CA 92093, U.S.A.; †Center for Drug Discovery Innovation, University of California, San Diego, 9500 Gilman Drive, La Jolla, CA 92093, U.S.A.

**Keywords:** chemotherapy, translation regulation, UV cross-linking, X-ray crystallography, 5-FU, 5-fluorouracil, dUMP, 2′-deoxyuridine-5′-monophosphate, FdUMP, 5-fluoro-dUMP, ESI, electrospray ionization, hTS, human thymidylate synthase, IDA, independent data acquisition, IVT, *in vitro* translation, LC, liquid chromatography, MS/MS, tandem MS, PDPA, 1,3-propanediphosphonic acid, TFA, trifluoroacetic acid, THF, tetrahydrofolate, wt, wild–type

## Abstract

Expression of hTS (human thymidylate synthase), a key enzyme in thymidine biosynthesis, is regulated on the translational level through a feedback mechanism that is rarely found in eukaryotes. At low substrate concentrations, the ligand-free enzyme binds to its own mRNA and stabilizes a hairpin structure that sequesters the start codon. When in complex with dUMP (2′-deoxyuridine-5′-monophosphate) and a THF (tetrahydrofolate) cofactor, the enzyme adopts a conformation that is unable to bind and repress expression of mRNA. Here, we have used a combination of X-ray crystallography, RNA mutagenesis and site-specific cross-linking studies to investigate the molecular recognition of TS mRNA by the hTS enzyme. The interacting mRNA region was narrowed to the start codon and immediately flanking sequences. In the hTS enzyme, a helix–loop–helix domain on the protein surface was identified as the putative RNA-binding site.

## Abstract 2

We used crystallography and site-specific cross-linking to study interacting regions of the human thymidylate synthase and its cognate mRNA, which form a complex that represses translation of the enzyme and is implicated in resistance of tumours to antimetabolite chemotherapy.

## INTRODUCTION

Cellular DNA synthesis critically depends on the supply of nucleotide triphosphate building blocks. The only *de novo* biosynthetic pathway to generate dTTP (2′-deoxythymidine-5′-triphosphate) requires reductive methylation of dUMP (2′-deoxyuridine-5′-monophosphate) to dTMP (2′-deoxythymidine-5′-monophosphate) by TS [1]. The TS enzyme is an obligatory homodimer [2] whose subunits associate with nanomolar affinity [3] to form a dimer that adopts an asymmetric conformation upon substrate binding [4,5]. Inhibition of TS leads to the cessation of DNA replication and thymineless death of proliferating cells [6], which renders the enzyme an attractive target for cancer chemotherapy [7]. TS inhibitor drugs include 5-FU (5-fluorouracil), which was one of the earliest anti-cancer agents and is still used in the treatment of colorectal cancer [8,9]. 5-FU is metabolized to FdUMP (5-fluoro-dUMP) which covalently modifies the TS active site, forming a ternary complex that also contains the methylene-THF (tetrahydrofolate) cofactor [7]. Other drugs that target TS, for example raltitrexed, compete directly with the binding of the THF cofactor [10].

The clinical use of TS inhibitors is limited by emerging tumour resistance which arises from an increase in TS protein levels. Among the mechanisms leading to rising the TS levels are reduced turnover and increased the stability of the protein in the presence of enzyme–inhibitor complexes and the up-regulation of TS expression [6,7,11–13]. The increase in TS expression occurring during 5-FU chemotherapy has been associated with an autoregulatory mechanism of translation control for the enzyme [14]. Ligand-free TS protein binds its own mRNA and thereby represses translation [15,16]. Complex formation with the dUMP substrate or inhibitors, including FdUMP abolishes mRNA binding of TS [17]. Therefore increased levels of TS expression are observed during chemotherapy with 5-FU despite inactivation of the enzyme, which ultimately results in emergence of tumour resistance. Feedback regulation by protein binding to mRNA is a common mechanism of translational regulation in bacteria, but rare in eukaryotes. The TS system represents the first known example of translational autoregulation in human [18].

**Figure 1:**
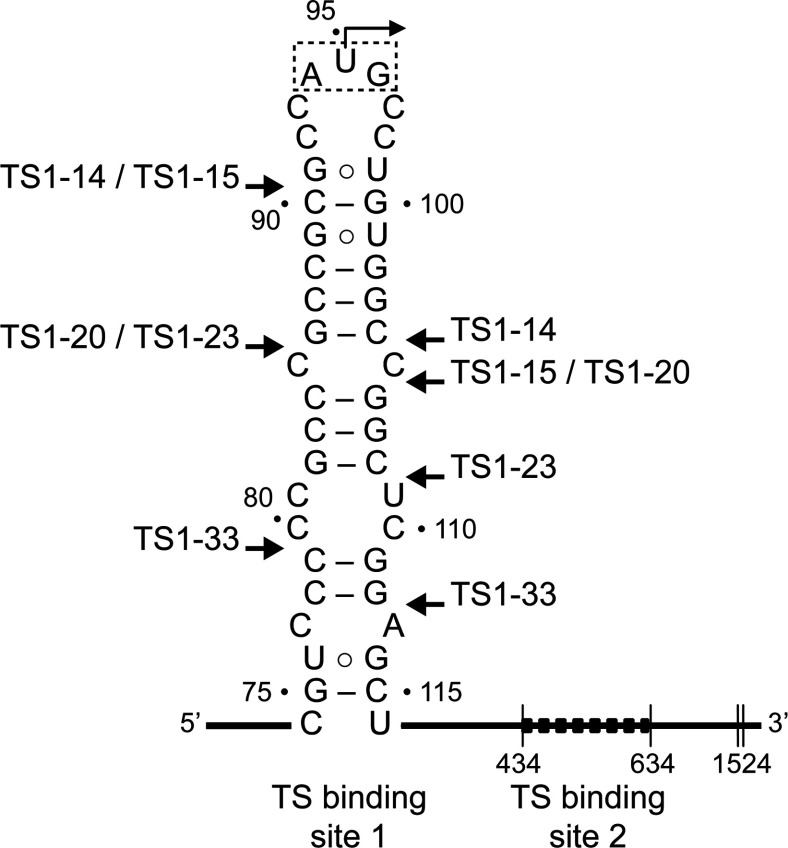
Secondary structure of the TS1 (thymidylate synthase-binding site 1) in the mRNA of the human enzyme TS binding sequesters the AUG initiation codon. A second TS-binding site is located within the reading frame. Arrows mark the boundaries of truncated TS1 motifs (TS1-X, where X indicates the length) which were used for *in vitro* translation, co-crystallization and cross-linking experiments described in this study. Numbering is according to the *Homo sapiens* sequence, record NM_001071 of the NCBI Nucleotide Database.

In the TS system, full translational repression is caused by protein binding at two mRNA sites [16]. One of the TS-binding sequences (site 2) resides in an extended region of 200 nucleotides in the mRNA-coding region. The site 1 is predicted to fold into a stem loop structure that contains the translation initiation site (Figure 1) [15]. TS protein binding to the regulatory mRNA site 1 motif likely stabilizes the hairpin loop that renders the start codon unavailable for ribosomal recognition. In a previous investigation we had demonstrated that the TS site 1 hairpin constitutes an autonomous regulatory RNA motif that maintains its function when transplanted into heterologous reporter systems [19]. From mutational and mechanistic studies of the TS site 1 motif we concluded that secondary structure of the RNA by itself provides only a marginally stable roadblock to ribosomal initiation, whereas binding of the TS protein reduces translation initiation by sequestration of the start codon. Here, we have used a combination of X-ray crystallography, *in vitro* translation functional studies and UV cross-linking to investigate the molecular recognition of the TS site 1 RNA motif by the enzyme.

## EXPERIMENTAL

### Reagents

Restriction nucleases, ligases and competent cells were from New England Biolabs, plasmid purification kits from Promega and restriction digest clean-up kits from Qiagen. Ni Sepharose 6 Fast Flow was from General Electric. Salts, 2-mercaptoethanol, 37% (v/v) formaldehyde, imidazole, DTT, THF, Tris and acetonitrile were from Sigma. CHAPS was from Thermo Fisher Scientific. LB (Luria–Bertani) broth extract was from Becton, Dickinson and Company. IPTG (isopropyl β-D-thiogalactopyranoside) and ampicillin were from Research Products International. 5-FU was from MP Biomedicals. Amicon Ultra-15 Centrifugal Filter Units were from Millipore. All experiments were conducted in ultra-pure RNase-free water.

### Nucleic acids and hTS (human thymidylate synthase) protein

DNA oligonucleotides for cloning and TS site 1 RNA for binding studies as well as 4-thiouridine-modified RNA for cross-linking were chemically custom-synthesized by Integrated DNA Technologies. Hexahistidine tagged hTS protein was expressed in *Escherichia coli* and purified by chromatography over an Ni^2+^ affinity column as described previously [19]. Bacterial TS was removed by an additional wash step adding 10 column volumes of 1% (v/v) CHAPS buffer. The hTS protein was eluted on a linear gradient of 40–100 mM imidazole. Eluted fractions were run on an SDS–10%PAGE to ascertain purity. Fractions were pooled, concentrated and dialysed into 20 mM Tris pH 8, 1 mM DTT. The enzyme was stored for extended periods at −80°C.

### Native PAGE of protein–RNA complexes

Precast Expedeon PAGE (8% gel) were used for native PAGE analysis of hTS–RNA complexes. The electrophoresis buffer contained 50 mM Mops, pH 7.0 and 1 mM DTT. Samples were loaded in a 1:1 mixture with 2× native loading buffer containing 40 mM Mops, pH 7.0, 4 mM DTT and 4 mM MgCl_2_. RNA was visualized with SYBR® Gold nucleic acid stain.

### Crystallography, data collection and structure determination

Crystallization was performed by hanging drop vapour diffusion at room temperature (22°C) with hTS protein and TS1-33 RNA, pre-incubated together at 150 μM concentration, in the presence of either 1.75 M ammonium sulfate, 0.1 M Tris, pH 8.25, 20 mM 2-mercaptoethanol (‘high salt’) or 30% PEG [poly(ethylene glycol)] 1500 0.1M Tris, pH 8, 20mM 2-mercaptoethanol and 3% (w/v) 1,5-diaminopentane dihydrochloride (‘low salt’). The final drop volume was 2–4 μl. Crystals appeared within 24 h, and grew to full size within 3–5 days. Crystals were flash-frozen in liquid nitrogen before data collection. Diffraction data were collected in a nitrogen stream at 110 K on a Rigaku MicroMaxTM 007 HF rotating anode X-ray generator (λ=1.54 Å) equipped with VariMax optics and a MAR345 imaging plate detector system. Datasets collected were processed, integrated and scaled with the HKL2000 package [20]. Structures were solved by molecular replacement with the program Phaser [21] using search models derived from published hTS coordinates [22,23] and refined by the program Refmac [24] both within the CCP4 package [25]. Subsequent iterative rounds of manual building and refinement, alternating between Refmac and manual rebuilding in Coot [26], were based on the obtained 2F_o_–F_c_ and F_o_–F_c_ maps. Positions of sulfate ions were initially assigned based on electron density as well as geometry of coordinating ligands. Final refinement was carried out in PHENIX [27] with individual isotropic atomic displacement parameters and water picking (Supplementary Tables S1 and S2). Coordinates and structure factors for the hTS protein have been deposited in the RCSB PDB under accession codes 4H1I (low salt structure) and 4GYH (high salt structure).

### Bicistronic reporter constructs for IVT (*in vitro* transcription)–translation

Cloning of bicistronic reporter constructs TS1-wt (wild-type) and ∆TS1 (Supplementary Figure S4) was performed as described previously [19]. The TS1-wt construct was used as the starting material for preparation of the TS1-xx truncation mutants, whose sequences are shown in Figure 1, by QuickChange Mutagenesis according to manufacturer's protocol (Agilent). Mutations were introduced by synthetic oligonucleotide primers that contained the desired truncated TS1 hairpin and 12 complementary nucleotides upstream and downstream of the mutation site. The non-complementary sequences inserted into the respective constructs are listed in the Supplementary Table S5. The integrity of reporter constructs carrying TS1-xx truncation mutants was confirmed by sequencing.

### IVT-translation assay and luciferase detection

The IVT assay using bicistronic luciferase reporter constructs (Figure 3) was performed as described previously [19]. For experiments in the presence of hTS, protein was added from stock solution to achieve a final concentration of 5 μM in the IVT reaction mixture. No other modifications were made.

### Cross-linking of hTS–RNA complexes

The cross-linking of the hTS protein to the TS1–33 and TS1–14 RNAs was conducted at room temperature (22°C). Concentrations of the protein and the RNA were 200 μM each in the initial cross-linking experiments (Figures 4A and 4B), and 25 μM protein with 10 μM RNA in the time course analysis (Figure 4C). The final buffer conditions contained 10 mM Hepes, pH 7.0, 100 mM NaCl, 2 mM DTT and 10% (v/v) glycerol. The hTS–RNA complex was kept on ice for 5 min prior to exposure to UV light at a wavelength of 360 nm. After UV exposure of 10 min (Figures 4A and 4B) or the designated time points in the kinetic analysis (Figure 4C), samples were conserved on ice until their subsequent loading on a polyacrylamide gel. After electrophoretic separation for 30–60 min, the protein was visualized using SimplyBlue SafeStain (Invitrogen). As a control, samples of hTS in the absence of RNA were cross-linked and analysed identically.

### Protease digestion of cross-linked hTS–RNA complexes

MS analysis of peptide fragments was performed following established procedures [28]. Slices of PAGE from electrophoretic separation containing cross-linked complex, or free hTS as a control, were cut to 1 mm×1 mm cubes and destained three times by first washing with 100 μl of 100 mM ammonium bicarbonate for 15 min, followed by addition of the same volume of acetonitrile for 15 min. The supernatant was removed and samples were dried in a speedvac. Samples were then transferred to reducing conditions by mixing with 200 μl of 100 mM ammonium bicarbonate and 10 mM DTT, and incubated at 56°C for 30 min. After removal of the liquid, 200 μl of 100 mM ammonium bicarbonate and 55 mM iodoacetamide was added to gel pieces and incubated at room temperature (22°C) in the dark for 20 min. After the removal of the supernatant and one wash with 100 mM ammonium bicarbonate for 15 min, an equivalent volume of acetonitrile was added to dehydrate the gel pieces. The solution was then removed and samples were dried in a speedvac. For protease digestion, enough solution of ice-cold trypsin (0.01 μg/μl) in 50 mM ammonium bicarbonate was added to cover the gel pieces, and then kept on ice for 30 min. After complete rehydration, the excess trypsin solution was removed, replaced with fresh 50 mM ammonium bicarbonate, and left overnight at 37°C. Peptides were extracted twice by the addition of 50 μl of 0.2% (v/v) formic acid and 5% (v/v) acetonitrile followed by vortexing at room temperature (22°C) for 30 min. The supernatant was removed and preserved. A total of 50 μl of 50% acetonitrile–0.2% formic acid was added to the sample, which was vortexed again at room temperature for 30 min. The supernatant was removed and combined with the supernatant from the first extraction. The combined extraction solutions were analysed directly by HPLC) coupled with MS/MS (tandem MS) using ESI (electrospray ionization). As a control, UV-irradiated samples of hTS in the absence of RNA were analysed identically.

### MS analysis of proteolytic peptides from cross-linked hTS–RNA complexes

ESI of peptide fractions was performed using a TripleTof 5600 hybrid mass spectrometer (ABSCIEX) interfaced with nano-scale RP-HPLC (reverse-phase HPLC) (Tempo) using a 10 cm–100 μm ID glass capillary packed with 5 μm C18 Zorbax™ beads (Agilent). Peptides were eluted from the C18 column into the mass spectrometer using a linear gradient (5–60%) of acetonitrile at a flow rate of 250 μl/min for 1 h. The buffers used to create the acetonitrile gradient were: buffer A, 98% H_2_O, 2% acetonitrile, 0.2% formic acid and 0.005% TFA (trifluoroacetic acid); buffer B, 100% acetonitrile, 0.2% formic acid and 0.005% TFA. MS/MS data were acquired in a data-dependent manner in which the MS1 data were acquired for 250 ms at *m*/*z* of 400–1250 Da and the MS/MS data were acquired from *m*/*z* of 50–2000 Da. The IDA (independent data acquisition) parameters were MS1-TOF (time-of-flight) 250 ms, followed by 50 MS2 events of 25 ms each. The IDA criteria were, over 200 counts threshold, charge state +2–4 with 4 s exclusion. The collected data were analysed using MASCOT® (Matrix Sciences) and Protein Pilot 4.5 (ABSCIEX) for peptide identification. Peakview (ABSCIEX) was used for peptide quantification analysis. For this analysis the *m*/*z* corresponding to each peptide was extracted from the MS1 chromatogram and the area under the peak was determined. Peak areas for the peptides that showed no change in their relative area were used to normalize different chromatograms analysed in these experiments. As a control for peptide abundance, samples of hTS UV-irradiated in the absence of RNA were analysed identically.

## RESULTS

### Crystallization and structure determination of hTS in the active and inactive conformation

**Figure 2:**
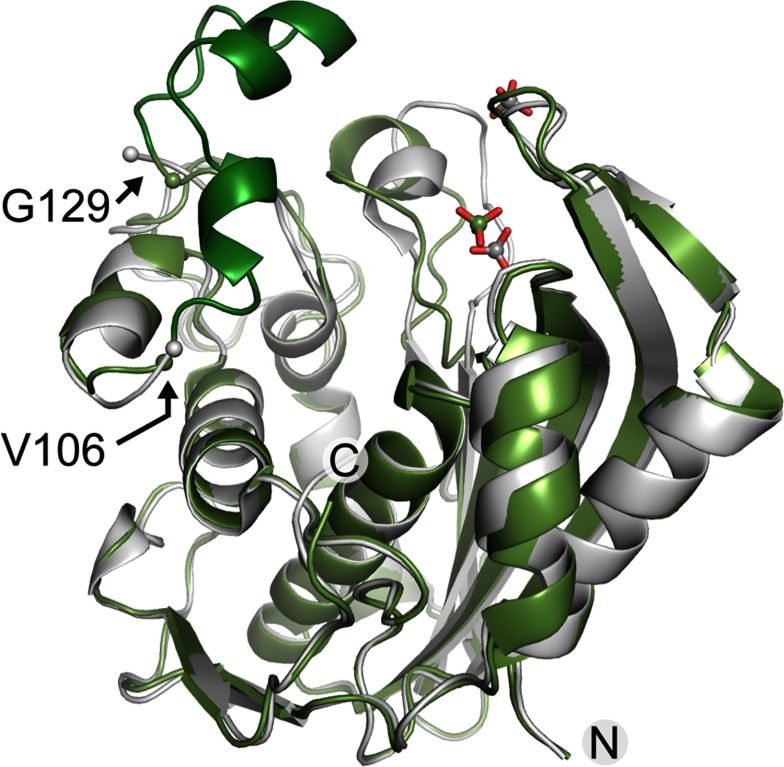
Superposition of hTS structures The protein structures were determined by X-ray diffraction from crystals grown at low (green, PDB code 4H1I) and high (grey, PDB code 4GYH) salt conditions. Only one subunit of the hTS dimer is shown. The K107–E128 peptide, which is disordered in the high salt structure, is highlighted in dark green in the low salt structure. The flanking ordered residues V106 and G129 are indicated by spheres in both structures. Two sulfate ions which are part of the high salt structure are shown in grey, one sulphate in the low salt structure is shown in green.

To gain direct insight into the molecular recognition of mRNA by hTS, we attempted co-crystallization of the enzyme with oligonucleotide constructs representing the TS site 1 motif (Figure 1). Diffracting crystals were obtained at varying salt conditions for hTS in the presence of the 33 nt RNA construct TS1-33 which binds the hTS protein (Supplementary Figure S1. The TS1–33 RNA covers nucleotides 80–112 of the TS site 1 motif and largely overlaps with a 36 residue oligonucleotide (‘35-TS’) spanning the sequence of positions 75–110, which is a previously reported minimal oligonucleotide construct shown to bind hTS [15]. X-ray data sets were collected and structures determined for crystals grown at low salt (100 mM buffer) as well as high salt (1.75 M ammonium sulphate) concentration (Supplementary Tables S1 and S2). Molecular replacement with previously published hTS coordinates furnished structures with the enzyme adopting either of the previously observed active and inactive conformations of the loop regions about I108-G129 and A181-A197 (Figure 2 and Supplementary Table S3); however, each without RNA bound. In the active conformation, the catalytic C195 is located in the active site, while it resides at the dimer interface in the inactive conformation. The structure of hTS obtained at high salt (PDB: 4GYH) was nearly identical to a previously published crystal structure of the enzyme in the inactive conformation in a complex with the allosteric inhibitor PDPA (1,3-propanediphosphonic acid) (PDB: 2ONB) [23]. The hTS structure determined at low salt (PDB: 4H1I) represents the first time that the ligand-free protein has been captured in the active conformation, which was previously observed only in a mutant containing the stabilizing mutation R163K (PDB: 2RD8) [29] and in substrate complexes, including a ternary complex of the enzyme with dUMP as well as the folic acid analogue inhibitor raltitrexed (PDB: 1HVY) [30]. The structure of the hTS active conformation in 4H1I is essentially identical to the structure of the R163K variant (Supplementary Figure S2 and Supplementary Table S3). In both the low and high salt structures 4GYH and 4H1I, a sulfate ion was located at the position of the dUMP phosphate group in the ternary complex. A second sulfate was present in the high salt structure coinciding with the location of a phosphate from the PDPA inhibitor in the published inactive conformation (PDB: 2ONB) [23]. Apart from the previously observed conformational distinction around the loop I108-G129, the peptide K107-E128 was disordered in the high salt structure but adopted a well-defined helix–loop–helix motif in the low salt structure (Figure 2) as previously observed in the R163K mutant [29] and the ternary complex (PDB: 1HVY) [30].

The structures of hTS observed in the crystals grown at low and high salt conditions do not reveal why the TS1-33 RNA failed to co-crystallize with the protein. Previously, it had been proposed that hTS functions as a metabolic enzyme in the active conformation but as a nucleic acid-binding protein in the inactive state [12]. Structure comparison of native and substrate-liganded hTS suggested that nucleic acid-binding might be achieved in the inactive but not the active conformation of the enzyme [22,30]. Inspection of the crystal structure of hTS in the active state, which we obtained at low salt conditions (PDB: 4H1I), revealed that packing of the protein dimers in the crystal involves the surface including a helix–loop–helix motif around residues G84-K104. As outlined below, cross-linking studies with TS site 1 RNA indicate that the same helix–loop–helix motif provides the nucleic acid-binding site of hTS (Supplementary Figure S3). Crystallization of hTS at low salt may have led to discrimination against RNA ligand binding by crystal contacts that involve the putative RNA-binding region.

### The hTS enzyme recognizes the apical hairpin of the TS site 1 mRNA motif

**Figure 3:**
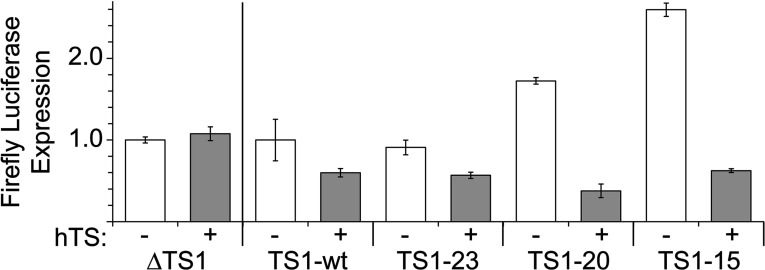
Luciferase reporter translation under the control of the TS1 regulatory element Translation levels are shown for the wt TS1 element (TS1-wt) and truncated versions (TS1-23, 20, 15) in the absence and presence of the hTS enzyme. As a comparison, the expression is shown from a construct lacking the TS1 motif (∆TS1). Construct structures are shown in the Supplementary Figure S3. For experiments in the presence of hTS (+) the recombinant enzyme was added at 5 μM concentration. Expression from TS1 constructs (TS1-23, 20, 15) in the absence of protein (−, white columns) was normalized to the wt motif (TS1-wt) to demonstrate the increase of translation efficiency with decreasing stability of the TS1 truncation variant. Expression in the presence of hTS (+, grey columns) was normalized for each TS1 construct to its own translation efficiency in the absence of protein to indicate the relative decrease caused by hTS binding on a scale from 0 to 1. Data are averages of three replicates with error bars showing ±1σ.

To better define the binding site of hTS on the mRNA motif, we investigated the function of TS site 1 truncation mutants in which parts of the stem region were deleted. The impact of the deletions was studied using a coupled IVT-translation assay that measured protein expression by quantitation of luminescence from a luciferase reporter. A previously established bicistronic construct carrying firefly luciferase under the control of the TS site 1 motif and an internal ribosome entry site-driven *Renilla* luciferase as a TS-independent internal standard [19] (Supplementary Figure S4) was modified to furnish three stem-truncation mutants, including TS1–23, TS1–20 and TS1–15 (Figure 1). Truncations were chosen to maintain the pyrimidine-rich flanking sequences around the initiation codon as well as the reading frame of the luciferase reporter. The T1–15 construct was the shortest variant that still maintained a hairpin secondary structure around the start codon. As we had demonstrated previously, addition of hTS inhibits reporter expression from the TS1-wt construct because of the protein binding to the TS site 1 RNA motif and thereby sequestering the start codon [19]. Expression from a control construct, ∆TS1, which does not contain the TS regulatory motif, is unaffected by the presence of hTS. As expected, translation efficiency of the truncation mutants in the absence of hTS increased relative to TS1-wt in correlation with the lower stability of the truncated variants, illustrating how less stable hairpin secondary structures present a smaller energy barrier to overcome by the translation machinery. However, in the presence of the hTS protein, all three TS1 truncation mutants showed reduced reporter expression (Figure 3). This suggests that sequences immediately flanking the start codon in the apical hairpin loop of the TS site 1 RNA are sufficient for interaction with the hTS protein, while the lower stem region may play a subordinate role. Importantly, the C85–C105 mismatch (Figure 1), which has been proposed as a key determinant of small molecule ligand binding to the TS site 1 RNA [31,32], is not an absolute requirement for hTS binding as none of the truncation mutants contained this motif. This is consistent with our previous finding that a TS site 1 RNA mutant in which the C85–C105 mismatch was replaced by a G85–C105 base pair was still able to bind the hTS protein [19].

### Site-specific cross-linking reveals the site 1 RNA binding site in hTS

**Figure 4:**
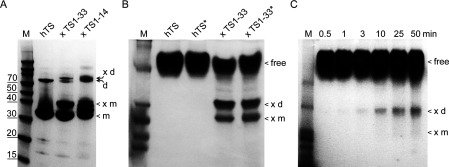
Cross-linking of hTS protein with TS1 RNA constructs in which the start codon U95 was substituted by 4-thiouridine (**A**) Denaturing PAGE analysis of cross-linked complexes with TS1-33 and TS1-14 RNA (for RNA sequences see Figure 1). Positions are indicated of the hTS monomer and dimer in the free form (m, d) and cross-linked (×m, ×d). Protein dimer may be present due to incomplete denaturation (see Supplementary Figure S5). (**B**) Native PAGE analysis of hTS cross-linked with TS1–33 RNA. Both protein and RNA were at 200 μM concentration. Free protein, cross-linked dimer (×d) and monomer (×m) are indicated. Conditions marked by * contain 1% zwitterionic detergent (CHAPS). (**C**) Native PAGE analysis of a cross-linking time course of hTS protein and TS1–33 RNA. Protein and RNA concentrations were 25 and 10 μM, respectively. Free protein, cross-linked dimer (×d) and monomer (×m) are indicated.

To identify the interacting regions in the complex of hTS and TS site 1 RNA, we performed cross-linking experiments guided by IVT studies of RNA truncation mutants, which suggested that the start codon and immediately flanking sequences may provide a binding site for the protein. Two RNA constructs, including the TS1–33 oligonucleotide used before in crystallization studies and a shorter TS1–14 oligonucleotide (Figure 1), were chemically synthesized with the U95 residue in the start codon replaced by 4-thiouridine [33]. Cross-linking was performed by UV-irradiation of hTS in complex with the thiouridine-labelled oligonucleotides. To limit covalent modification to thiouridine residues and avoid non-specific cross-linking, UV at a wavelength of 360 nm was used. Gel electrophoretic analysis of cross-linked samples showed that the longer TS1–33 construct formed a single defined complex with the hTS protein, while the TS1–14 oligonucleotide gave rise to several distinct complexes, likely because of multiple binding events of the smaller RNA (Figure 4A). It is conceivable that 1:1 complex formation with the TS1–33 RNA induces a conformational change in the hTS dimer such that binding of the cognate RNA motif at one subunit prevents the other protein monomer from associating with RNA. A similar effect had been observed in substrate and cofactor binding which occur with negative co-operativity by the two subunits [34]. The majority of crystal structures reported for TS from human and other organisms show a symmetric arrangement of subunits [35,36] which does not support half-of-the-sites reactivity. However, half-site occupation with ligands had been observed in structures of the *Pneumocystis carinii* TS and the human TS mutant R163K [4,5]. The proposed half-site binding of the cognate RNA ligand would be in agreement with the observation of a single cross-linked complex for the TS1–33 RNA and may explain why, even in the presence of excess TS1–33 RNA, the cross-linking efficiency never exceeded 50% of the available TS protein. Gel-electrophoretic analysis of complex from cross-linking of hTS protein with TS1–33 RNA under native conditions (Figure 4B) demonstrates that formation of the protein–RNA complex is compatible with both the hTS monomer and dimer, although dimer was present in excess. Kinetic analysis by following cross-linking over time suggested that the TS1–33 construct preferably cross-linked with the hTS dimer (Figure 4C) which is the dominating form of hTS in solution [3].

To identify domains of the hTS protein involved in TS1–33 RNA binding, we performed a qualitative MS analysis of peptide fragments from the cross-linked protein. Cross-linked hTS–TS1–33 complex in cut-out PAGE slices was digested by trypsin. Peptide fragments from the protease treatment were separated by LC (liquid chromatography) and identified with MS/MS. As a control, hTS protein in the absence of RNA was UV-irradiated under the same conditions as used for cross-linking and analysed identically to the cross-linked samples. Abundance of peptide fragments was calculated from the LC–MS/MS analyses and compared with the unliganded protein and the cross-linked complex (Supplementary Table S4). Peptides that were detected at a reduced rate in the cross-linked complex correspond to hTS domains that were either covalently modified by cross-linking with the TS1–33 RNA or that were protected from trypsin digestion because of conformational changes induced by RNA binding (Supplementary Figure S6). We did not detect mass signals corresponding to peptides that were modified by RNA cross-linking, however, and had to rely on observation of the abundance differential to identify potential interaction sites. The absence of signals for peptide–RNA conjugates was attributed to low ionization efficiency of then negatively charged species.

**Figure 5:**
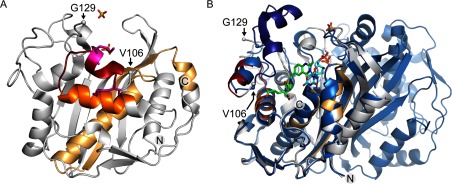
Results from TS site 1 RNA cross-linking with hTS Cross-linking results were mapped on the protein crystal structure determined at high salt conditions (grey, PDB code 4GYH) which corresponds to the RNA-binding inactive conformation of the enzyme. (**A**) Front view: MS analysis of proteolytic fragments from the cross-linked hTS–RNA complex shows that cross-linking occurs at a helix–loop–helix motif adjacent to the active site cleft. In the cross-linked complex, a peptide spanning the outer helix (G94–K104, red) was undetectable and the abundance of the peptide involved in the inner helix (G84–K93, orange) was about 150-fold reduced. Two lysine residues in the outer helix (K99, K104), which are likely cross-linking candidates, are highlighted in magenta stick representation. Abundances of peptides in adjacent helices and a β-sheet in the core of the protein were reduced as well (light orange), likely through indirect effects by RNA cross-linking at the neighbouring helix–loop–helix motif. (**B**) Side view: superposition of hTS in the active conformation (blue, PDB code 1HVY) which had been crystallized in complex with the folic acid analogue inhibitor raltitrexed (green) and dUMP substrate (cyan) covalently bound at the active site residue C195. Only one subunit is shown for 4GYH, the dimer for 1HVY. Two sulfate ions shown are part of the 4GYH structure. The K107–E128 peptide is disordered in the inactive conformation but well-ordered in the active conformation (dark blue). The flanking ordered residues V106 and G129 are indicated by spheres in both structures. Two carboxylate groups of raltitrexed are positioned in close proximity to the G84–K93 helix which cross-links to RNA.

The cross-linking and MS analysis of peptide fragments was performed in triplicate and peptide abundance observations were considered that occurred consistently in all three experiments. When peptides with significantly decreased abundance in the complex were mapped on the hTS crystal structure, a helix–loop–helix motif (G84–K104) adjacent to the active site cleft was identified as the likely cross-linking domain (Figure 5). In the cross-linked complex, a peptide involved in a solvent-exposed helix (G94–K104) was undetectable, and the abundance of a preceding peptide involved in a second helix (G84–K93) was reduced by about 150-fold. Abundances of peptides in adjacently packing helices and a β-sheet in the core of the protein were reduced as well, likely through indirect effects by RNA cross-linking at the neighbouring helix–loop–helix motif. The G84–K104 helix–loop–helix domain is located at the periphery of the hTS fold with the G94–K104 helix protruding from the protein surface. The helix involving residues G84–K93 has one face exposed to solvent, while the other side provides a wall of the active site cleft. The accessibility of the helix–loop–helix motif on the protein exterior and cross-linking data are consistent with a proposed function as the hTS RNA-binding site. However, the sequence of the G84–K104 helix–loop–helix domain bears no homology to known RNA-binding motifs, suggesting that hTS recognizes the TS site 1 RNA via a unique structural element. Deeper insight into details of the protein–RNA interaction will require a crystal structure of the complex.

## DISCUSSION

Here, we have used a combination of X-ray crystallography, RNA mutagenesis and site-specific cross-linking studies to investigate the molecular recognition of the site 1 motif in TS mRNA by the hTS protein. Although the crystallography effort furnished structures of the protein in both the active and inactive conformation, co-crystallization of an hTS–RNA complex remained elusive. Mutagenesis studies of the TS site 1 motif in the context of *in vitro* translation experiments and cross-linking of hTS–RNA complexes allowed narrowing the interacting RNA region to the start codon and immediately flanking sequences, mapping to the apical loop of the TS site 1 RNA motif. In the hTS enzyme, the helix–loop–helix domain spanning residues G84–K104 on the protein surface was identified as the putative binding site for TS site 1 RNA. Crystal structure analysis revealed that in the cofactor and dUMP substrate-bound, active conformation of the enzyme the G84–K104 helix–loop–helix motif is obstructed by the K107–E128 peptide which adopts an ordered, predominantly helical structure which may prevent RNA binding. In the inactive conformation, the K107–E128 peptide is disordered and allows ligand access to the G84–K104 helix–loop–helix motif. Interestingly, while TS is highly conserved among eukaryotes and prokaryotes, an insertion of 12 residues within the K107–E128 peptide (at position 117) and a second insertion of eight residues at position 148 are unique to eukaryotes and only the human enzyme has been found to bind its own mRNA at the initiation site [1,37].

Without detailed insight into the structure of the protein–RNA complex, it is not clear how RNA binding to one subunit affects the other subunit in the hTS dimer. However, results from the present study that led to the identification of the putative RNA-interaction domain will guide future research including mutational analysis of the helix–loop–helix motif in G84–K104 and the conformationally flexible K107–E128 peptide. Quantitative optimization of the hTS–RNA cross-linking approach outlined here promises to provide covalently tethered complex for future crystallization and X-ray diffraction studies, which will eventually yield a high-resolution picture of the hTS–RNA complex.
